# Giant Congenital Sialolipoma of Parotid Gland with Parapharyngeal Extension

**Published:** 2019-03

**Authors:** Taner-Kemal Erdağ, Yüksel Olgun, Melih-Arif Közen, Handan Güleryüz, Erdener Özer

**Affiliations:** 1 *Department of Otorhinolaryngology, Dokuz Eylül University School of Medicine, Izmir, Turkey.*; 2 *Department of Radiology, Dokuz Eylül University School of Medicine, Izmir, Turkey.*; 3 *Department of Pathology, Dokuz Eylül University School of Medicine, Izmir, Turkey. *

**Keywords:** Congenital, Parotid, Sialolipoma

## Abstract

**Introduction:**

Sialolipoma is an extremely rare salivary gland tumor characterized by a well circumscribed mass composed of glandular tissue and mature adipose elements. Herein our aim was to present the fifth case of congenital sialolipoma, which was firstly followed up as a parotid gland hemangioma, and underline the fact that sialolipomas should be kept in mind in the differential diagnosis of congenital parotid gland masses.

**Case Report:**

A 10-month old male presented with a left-sided huge neck mass which progressed after birth. Radiologic examination revealed a tumor originating from the parotid gland filling the parapharyngeal space. Histopathologic examination of an incisional biopsy was consistent with sialolipoma. A total parotidectomy with preservation of the facial nerve was performed at the age of 1 year. The postoperative recovery was uneventful with normal facial nerve function. There was no recurrence at the 24-month follow-up.

**Conclusion:**

Although it is a very rare benign tumor, congenital sialolipoma should be kept in mind in the differential diagnosis of congenital parotid mass.

## Introduction

Sialolipoma was firstly described by Nagao et al. as a new type of benign salivary gland neoplasm characterized by a well circumscribed mass composed of glandular tissue and mature adipose elements (1). Although commonly seen in the parotid gland, sialolipoma may also arise from submandibular gland, as well as minor salivary glands (2–8). It constitutes only 0.3% of all salivary gland tumors in adults, and more than 40 cases of sialolipoma have been reported in the English literature (1,3). Congenital sialolipoma is an extremely rare tumor, and the first case was reported by Hornigold et al. in 2005 in a 7-week-old infant as a parotid gland mass (2). Three more congenital sialolipoma cases arising from the parotid gland have subsequently been described (2–4).

Herein our aim was to present the fifth case of congenital sialolipoma, which was firstly followed up as a parotid gland hemangioma, and underline the fact that sialolipomas should be kept in mind in the differential diagnosis of congenital parotid gland masses.

## Case Report

**Fig 1:**
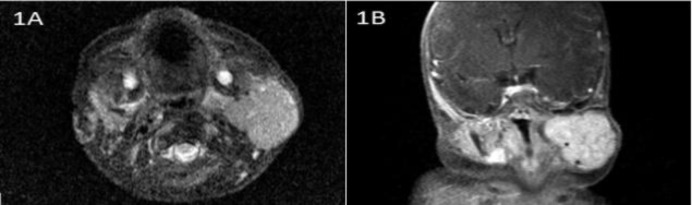
a) Neck MRI, T2-weighted axial image; b) contrast enhanced image showing left-sided hyperintense mass lesion with contrast enhancement

A 3-month-old male was seen in our pediatric oncology department because of a growing parotid gland mass. The mass was congenital and was followed by the pediatric oncology team with a prediagnosis of hemangioma. The patient’s magnetic resonance imaging (MRI) scan showed an infantile hemangioma in the proliferative phase filling the parapharyngeal space (Fig.1a,b). 

**Fig 2:**
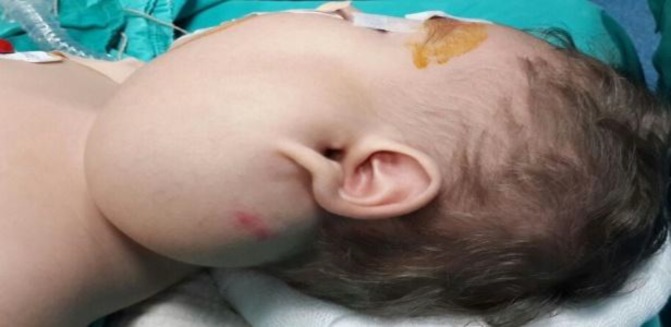
Patients preoperative otorhinolaryngological examination revealed an 8 x 7 cm mass filling the left parotid region

The child then received propranolol therapy, but despite this treatment the mass continued to grow, and the child was referred to our department at the age of 10 months. The otorhinolaryngological examination revealed an 8×7-cm mass filling the left parotid region, with normal facial nerve motor functions (Fig.2). Histopathological diagnosis of the mass was provided by an incisional biopsy as sialolipoma.

**Fig 3:**
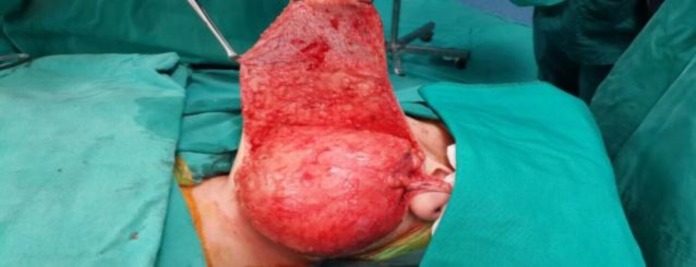
Giant mass arising from the parotid gland after elevation of the flaps

We planned to perform total excision of the mass with a transparotid approach. The facial nerve was monitored intra-operatively, and surgery was performed under magnification using surgical loops. A standard modified Blair incision was used, flaps were raised (Fig.3), then the facial nerve was identified in a standard fashion using a tragal pointer and posterior belly of the digastric muscle as landmarks. 

**Fig 4:**
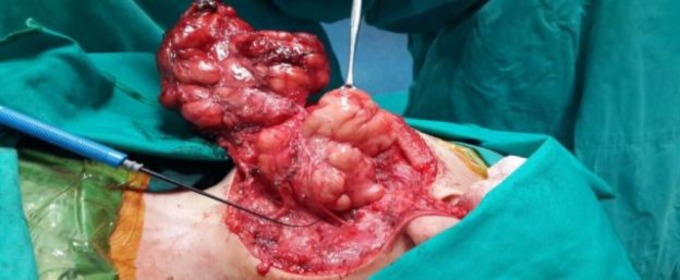
The main trunk of the facial nerve was found to be unusually elongated before the pes anserinus. After dissection of the portion of the tumor involving superficial parotid lobe, the deep lobe of the parotid gland and portion of the tumor filling the parapharyngeal space was encountered

The main trunk of the facial nerve was found to be unusually elongated before pes anserinus. A soft lobular mass was encountered. A tumor involving the superficial parotid lobe was first dissected, then the deep lobe of the parotid gland and portion of the tumor filling the parapharyngeal space were gently dissected under the facial nerve (Fig.4). A Jackson-Pratt drain was inserted into the wound after the excision of the tumor.

**Fig 5:**
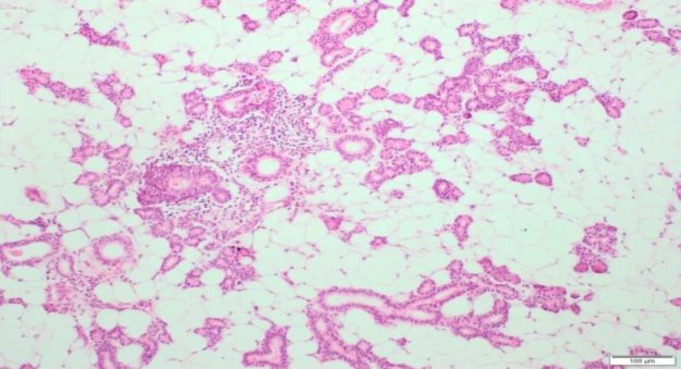
The lesion was composed of mature adipose tissue mixed with acinar and ductal structures of a salivary gland and lymphoid infiltrate. H&E staining, ×100

On macroscopic examination, the mass was found to be a lipomatous specimen, 9×8×4 cm in size. The cut surface was consistent with the appearance of a lipoma. Histopathologic examination confirmed the diagnosis of a sialolipoma. The lesion was composed of mature adipose tissue mixed with acinar, ductal structures of a normal salivary gland (Fig.5).

The postoperative period was uneventful. The patient’s facial nerve motor functions were well preserved, and he was discharged on the third postoperative day. The patient is still followed, and has no signs of recurrence in his 24th postoperative month.

## Discussion

The term sialolipoma was first used by Nagao et al. when they described seven sialolipoma cases in their retrospective analysis of the 2,051 primary salivary gland tumor specimens (1). In 2005, the term sialolipoma was accepted in the World Health Organization (WHO) classification of head and neck tumors (9). Sialolipoma is a mixed tumor consisting of salivary gland elements and mature adipocytes (1,2,9). Usually it is seen as a lobular tumor, which is similar to our case, and histopathologically it may show lobules with rich and fatty components surrounding the salivary elements (9). The majority of sialolipomas are seen in the parotid region, but the submandibular gland and other minor salivary glands may be the site of origin in some cases (2–8).

To date, more than 40 cases of sialolipomas have been presented, but the majority are in adult patients. Sialolipoma in children is extremely rare, with only four congenital cases depicted to date (2–5). To the best of our knowledge, our case is the fifth case of congenital sialolipoma. Interestingly, all congenital cases were derived from the parotid gland. In addition, two non-congenital sialolipoma cases arising from the submandibular and parotid glands have been described in two 3-year-old children (6,10). The most common congenital tumor of the parotid gland is hemangioma, although branchial cleft cyst, cystic hygroma, and sialoblastoma are among other congenital masses that can be seen in this period (2,4). Our patient presented with a rapidly growing congenital parotid swelling and was given an initial diagnosis of hemangioma in the proliferative phase based on MRI findings (increased signal on T2 weighted series and homogenous contrast enhancement of the mass). Despite propranolol treatment, the mass continued to grow, so we performed an incisional biopsy which was described as sialolipoma. The efficacy of MRI for differentiating sialolipoma from other congenital parotid gland masses is not adequate, but it can give information on the extent of the lesion (4). Fine needle aspiration biopsy is usually not helpful in cases of sialolipoma (3,4). Preoperative diagnosis can be obtained by US-guided core or incisional biopsies.

Parotidectomy in small children may present some difficulties. First of all, anatomical structures are much smaller, the facial nerve is located relatively superficially in comparison with adult patients, and the mastoid process is not fully developed. Consequently, operating under magnification with surgical loops or microscopes is mandatory, and facial nerve monitoring may facilitate identification of the nerve. In our case, the main trunk of the facial nerve was found to be 5 cm, unusually elongated before the pes anserinus. We believe that this finding was due to pressure and the stretching effect of the giant tumor. Similar surgical findings were also reported by Kidambi et al (4). 

Complete surgical excision (total or superficial parotidectomy) with facial nerve preservation is the treatment of choice, and no recurrence has been reported in the literature regarding congenital sialolipomas. Our case was also followed for 24 months without any signs of recurrence.

## Conclusion

Although very rarely encountered, congenital sialolipomas should be kept in mind for the differential diagnosis of congenital parotid masses. Definitive treatment of this tumor is obtained with superficial/total parotidectomy depending on the extent of the lesion.
